# Erucic Acid—Both Sides of the Story: A Concise Review on Its Beneficial and Toxic Properties

**DOI:** 10.3390/molecules28041924

**Published:** 2023-02-17

**Authors:** Agnieszka Galanty, Marta Grudzińska, Wojciech Paździora, Paweł Paśko

**Affiliations:** 1 Department of Pharmacognosy, Jagiellonian University Medical College, Medyczna 9, 30-688 Kraków, Poland; 2 Department of Food Chemistry and Nutrition, Jagiellonian University Medical College, Medyczna 9, 30-688 Kraków, Poland

**Keywords:** erucic acid, cardiotoxicity, cytotoxic, neuroprotective

## Abstract

Erucic acid (EA) is monounsaturated fatty acid (22:1 n-9), synthesized in the seeds of many plants from the *Brassicaceae* family, with *Brassica napus*, *B. rapa*, or *B. carinata* considered as its richest source. As the compound has been blamed for the poisoning effect in Toxic Oil Syndrome, and some data indicated its cardiotoxicity to rats, EA has been for decades classified as toxic substance, the use of which should be avoided. However, the cardiac adverse effects of EA have not been confirmed in humans, and the experiments in animal models had many limitations. Thus, the aim of this review was to present the results of the so far published studies on both toxic, and pharmacological properties of EA, trying to answer the question on its future medicinal use. Despite the ambiguous and relatively small data on toxic and beneficial effects of EA it seems that the compound is worth investigating. Further research should be particularly directed at the verification EA toxicity, more in-depth studies on its neuroprotective and cytotoxic properties, but also its use in combination with other drugs, as well as its role as a drug carrier.

## 1. Introduction

**Figure 1:**
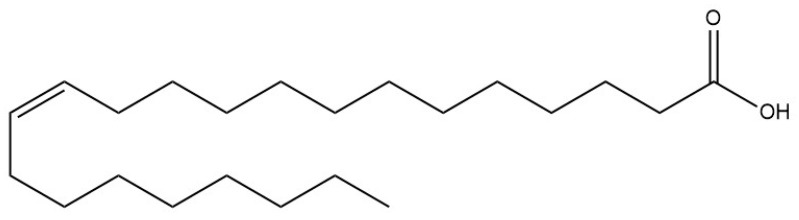
Structure of erucic acid.

Erucic acid (CH_3_(CH_2_)_7_CH=CH(CH_2_)_11_COOH) (Figure 1) is a 22-carbon monounsaturated fatty acid, with one double bond in the omega 9 position (22:1 n-9). The compound was named after the genus *Eruca*, but it is also synthesized in the seeds of other plants from the *Brassicaceae* family, of which some species and related varieties of *Brassica napus*, *B. rapa*, or *B. carinata* are its richest source (40–50% of the oil) [1]. The compound, however, can be also found in other oils, including sunflower oil (up to 900 mg/100 g), but also popular food products, e.g., cereals (up to 500 mg/100 g), pastries (up to 600 mg/100 g), salmon (up to 800 mg/100 g), or nuts (up to 300 mg/100 g) [2]. The seeds of *B. napus* are the source of one of the most popular edible oils, namely rapeseed oil, which for food purposes should be produced from ‘low-erucic acid rapeseed’ (LEAR) varieties. Such restrictions are due to the results of some in vivo studies, mainly published in the late 1970s, indicating that erucic acid (EA) may have cardiotoxic properties in rats [3]. Moreover, a severe poisoning with rapeseed oil, observed in over 20,000 people in Spain in 1981, known as a Toxic Oil Syndrome (TOS), was initially attributed to erucic acid (EA), although the results of further studies practically proved that the compound should not be blamed. The analysis of the toxic oil samples indicated low-quality olive oil, other vegetable oils, including rapeseed oil, and traces of aniline or oleoanilide. EA was present in the tested samples in very low concentrations; thus, the authors of the analysis excluded that the compound can be an etiologic agent responsible for intoxication [4,5]. Further WHO report concluded that the so far performed experiments in different animal models with the oil components suspected of the intoxication, including EA and its anilides or esters of 3-(*N*-phenylamino)-1,2-propanediol (PAP), did not confirm the involvement of these compounds in the pathogenesis of TOS [6].

However, the above-mentioned ambiguous data on EA toxicity caused a significant decrease in the studies performed on the pharmacological properties of this compound for almost a decade after the epidemic of TOS. The compound attracted scientific attention again in the early 1990s, when the first reports appeared on its use, in the form of so-called Lorenzo’s Oil (LO), a mixture of oleic acid and EA (4:1), in adrenoleukodystrophy (ALD). This is a rare, recessive X-linked disorder, resulting from a defect in β-oxidation of very long-chain fatty acids (VLCFA), and characterized by progressive cerebral demyelination. As EA has been a potent inhibitor of VLCFA synthesis, a number of clinical trials were then performed and their effects were well-summarized in a very recent systematic review, where four open trials (total *n* = 205) were included. The author concludes that LO may be effective at the early stages of ALD. Most importantly, no serious side effects were described, apart from the transient thrombocytopenia with no bleeding [7]. In recent years, the results of a number of studies indicated interesting pharmacological properties of EA or its use as a carrier for the transport of drugs. In the course of this review, we try to present the results of the published studies on the toxic and pharmacological properties of EA, trying to answer the following fundamental question: is there still a future for the medicinal use of EA?

## 2. Results

### 2.1. EA Toxic Effects

The strongest objection towards the use of EA was associated with its cardiotoxic potential. Some of the studies suggested that high, prolonged intakes of EA may be linked with the increase in fat deposits in heart muscle, called myocardial lipidosis. 3.

Heijkenskjöld et al. [3] evaluated the effects of EA (1.4 or 2.6 g/100 g of diet) on the oxidative metabolism of rat-heart mitochondria using intact animals, perfused beating hearts, isolated mitochondria, and mitochondrial extracts. EA diets led to a diminished ability of the isolated heart mitochondria to oxidize various substrates and accumulation of fat in the heart. The degree of the inhibition of biochemical processes was proportional to the EA content in the diet diminished upon prolonged rat feeding. Authors noted that even lower EA concentration used (1.4 g/100 g of diet) inhibited respiration with Krebs-cycle via erucyl carnitine, which inhibited the oxidation of other long-chain acyl carnitines but not that of glutamate, pyruvate, or succinate in heart mitochondria. What is important, this activity decreased after 8 weeks of the treatment. EA suppressed the rate of the oxidation of long-chain fatty acids, which may lead to an extramitochondrial accumulation of activated fatty acids and keep to increased triglyceride synthesis in the heart. The accumulated fat may thus affect the membranes and cause a general inhibition of mitochondrial respiration. On the other hand, Hulan et al. [8] compared the effect of rendered pig fat-fed diet, containing 20% rapeseed oil (22% EA) with a diet based on commercial lard, to which 5.4% free EA was added. Sprague-Dawley rats (*n* = 50) were used in this experiment which lasted 16 weeks. There were no significant differences observed in the level of EA in the hearts of rats fed with rendered pig fat-fed rapeseed oil, or commercial lard plus EA. However, the incidence and severity of cardiac lesions were significantly higher in rapeseed oil-fed rats. The results of this study indicate that the myocardial lesions associated with feeding 20% rapeseed oil diets are not related to the content of EA per se. Authors suggested that a triglyceride imbalance in the oil might play an important role in causing these lesions in rats.

The diet containing 5% EA influenced the metabolism of phosphatidylcholine in the hearts, but not in the livers, of rats (*n* = 15) after 5 weeks of treatment. The results indicated that the turnover of 1-stearoyl-2-arachidonoyl phosphatidylcholine in the heart was inhibited by the diet with EA. On the other hand, the proportion of EA in the free fatty acid fraction was higher in the heart than in the liver [9]. This was also confirmed two decades later by Murphy et al., who suggested that significant amounts of EA are metabolized in the liver to form saturated fatty acids, while in the heart EA remains more intact and it is targeted for pools destined for use in the heart for β-oxidation [10].

Long-lasting (up to 26 weeks) effects of a diet based on sunflower seed oil with the addition of rapeseed oil, or pure EA (8.9% *w*/*w*) were evaluated in rats (*n* = 18). The absence of less contractile reserve capacity effect was observed in the EA-treated animals. Additionally, the histological evaluation indicated no epicardiac fibrotic lesions. Authors suggested that EA is able to interfere with the contractile system of the peripheral vascular system. In the EA-treated group, the vasoconstrictor response toward norepinephrine was strongly reduced. Moreover, isoproterenol reduced myocardial contractility which has been attributed to a lowered perfusion pressure in the coronary blood supply of the myocardium with simultaneously increased energy demand. EA did not cause electrocardiographic changes in comparison with the control, untreated group. It was concluded that EA was not responsible for the loss of contractile reserve capacity without the changes in the myocardial conductance system. The authors also indicated that the combination of EA with linolenic acid might be the causative factor [11].

An interesting experiment performed in rats (*n* = 48) fed with EA (0.5% or 5%) and doxorubicin (2 mg/kg, i.p.), verified if EA prevents or augments the cardiotoxicity of the drug. Control animals were fed with standard pellets only (control) or with 2 mg/kg doxorubicin (control/DOX). During the 4 weeks experiment, the evaluation of malondialdehyde concentration, catalase, and cytochrome c oxidase activity and finally isolated heart measurements were performed. No significant difference was found between the groups during the study on the isolated heart for all evaluated parameters, except for dp/dtmax measured at baseline—it was significantly lower in the animals from the control/DOX group in comparison with the 0.5% EA group. No significant difference was found between groups for the level of MDA, catalase, and cytochrome c oxidase. The authors concluded that the EA diet increased doxorubicin toxicity, but when applied alone, neither doxorubicin nor EA showed a negative effect on survival and contractility [12]. On the contrary, Altinoz et al. [13] proved that the intraperitoneal co-administration of EA and doxorubicin (5 mg/kg + 100 mg/kg) to mice (*n* = 20) two times a week for 23 days, significantly decreased the toxic effects of the cytostatic drug in liver and heart tissues, when compared to the group receiving doxorubicin (5 mg/kg). The morphology of hepatocytes improved and was similar to the control, and so was the cardiac structure.

Two studies described the problem of the potential cardiotoxicity of EA in humans. Bierenbaum et al. [14] evaluated idiopathic cardiomyopathy among ten patients in Sichuan province, China, regularly consuming large amounts (500 mL/month) of mustard seed oil or rapeseed oil rich in EA. No correlation was observed between the high EA intake and degenerative cardiomyopathy among the patients. Contrary results were obtained in a prospective study, performed in two independent cohorts of 3694 older patients in the Cardiovascular Health Study (1992–2006) and of 3577 middle-aged patients in the Atherosclerosis Risk in the Communities Study (1987–2008) from Minnesota, US. A higher level of EA in plasma was positively correlated with the increased risk of congestive heart failure incidence in both cohorts, with a hazard ratio range of 1.34 to 1.92. However, the authors conclude that such an effect may result from the presence of other long-chain monounsaturated fatty acids, including nervonic acid circulating in the blood [15].

A brief summary of the studies on EA cardiotoxic effects is presented in Table 1.

Some data on EA toxicity other than cardiotoxicity comprised the in vitro studies referring to the impact of the compound on the cells of different origins. No toxic effect of EA was noted in rat lymphocytes at doses up to 100 µg/mL [16], while some studies indicated its toxicity to hepatocytes. At the concentration of 0.32 mM, EA caused a time-dependent decrease in hepatocyte viability after prolonged 96 h exposure, while the survived cells were enlarged, with more granular cytoplasm when compared to the untreated cells. Moreover, the increase in lactate dehydrogenase release was observed after 24 h incubation of hepatocytes with 0.32 mM of EA. Interestingly, a lower dose of EA (0.1 mM) revealed no effect on the activity of peroxisomal β-oxidation, carnitine acetyltransferase, or palmitoyltransferase [17]. Similarly, the compound not only increased β-oxidation activity in rat hepatocytes but was also cytotoxic to the cells, causing higher LDH leakage than in the untreated control cells [18]. On the contrary, no toxicity of EA to rat hepatoma Fao cells was observed, which may result from high peroxisomal activity in these cells [19]. In further parts of the experiment, the authors made an interesting observation on the selective EA toxicity to human fibroblasts, obtained from the patients with different peroxisomal disorders and multienzymatic deficiencies, namely Zellweger syndrome, neonatal ALD or infantile Refusum disease (IRD) and normal fibroblasts. The compound (25–150 µM) revealed toxicity only to the patients-derived fibroblasts, while normal cells were not affected, which was correlated with the absence of β-oxidation in the peroxisomes of the fibroblasts from the patients. EA was also differently accumulated in normal fibroblasts and those derived from IRD patients, with significantly higher uptake for the latter [19].

In some recent studies, EA did not inhibit the viability of mouse C3H10T1/2 pluripotent mesenchymal stem cells at concentrations up to 150 µM. The compound (25 µM) stimulated the differentiation of the cells rather into osteoblasts than adipocytes, by the inhibition of peroxisome proliferator-activated receptor (PPARɣ) transcriptional activity [20]. Moreover, the EA effect on human endoC-β-H1 cells was determined to understand the mechanism of lipotoxicity of beta-cell dysfunction connected with type 2 diabetes mellitus. EA (500 µM) highly stimulated apoptosis in the cells, with an increase in caspase-3 activity. The compound increased also the production of hydrogen peroxide generation in peroxisomes and mitochondria, accompanied by hydroxyl radical formation, cardiolipin peroxidation, and a decrease in ATP. Interestingly, EA did not induce the endoplasmic reticulum stress marker CHOP (C/-EBP homologous protein) gene [21].

### 2.2. EA Beneficial Effects

#### 2.2.1. Antibacterial and Antiviral Activity of EA and Its Derivatives In Vitro and In Vivo

EA revealed antibacterial activity in vitro against two *Borrelia* species: *B. burgdorferi* and *B. garinii*, with minimal bactericidal concentration MBC90 for stationary phase 0.75 and 0.70 mg/mL, respectively. The result was also performed for the logarithmic phase and MBC90 for both *Borrelia* species was 0.75 mg/mL and the activity was noted for both active and latent forms of bacteria [22].

Combinations of EA with other drugs were also investigated to verify if such an approach can increase the antimicrobial effect. In vitro studies of the newly formed conjugates of EA with ciprofloxacin showed however that the combination did not exert antimicrobial activity against *Staphylococcus aureus* (3 strains), *S. epidermidis*, *Escherichia hirae*, *E. coli* (2 strains), and *Pseudomonas aeruginosa* (2 strains), when compared to ciprofloxacin alone [23].

The interesting antiviral potential of EA, both in vitro and in vivo, was also described. The activity of the compound against five influenza A viral strains, expressed as IC_50_ was in the range of 0.49–1.24 mM, while the activity of oseltamivir, used as a positive control, was 1.22–9.93 µM. Moreover, the compound inhibited the activity of viral polymerase and also showed anti-inflammatory properties. As far as the molecular mechanism is concerned, EA inactivated NF-κB and p38 MAPK signaling pathways, which led to the reduction of transcriptional activity of interferon-stimulated gene factor 3 (ISGF3) and a decrease in pro-inflammatory responses. At the doses of 0.3–0.9 mM, EA also inhibited the apoptotic process stimulated by the influenza virus in human lung adenocarcinoma cells. The activity was subsequently confirmed by the in vivo study, on female BALB/c mice (*n* = 75) divided into a control group (non-infected), influenza virus (A/FM1/H1N1) infected group, and EA high (100 mg/kg/day) and low (50 mg/kg/day) dose treatment groups. EA, administered *i.g*. at 2 days prior to viral infection, consistently displayed decreased lung viral load and viral antigens expression. Meanwhile, EA markedly reduced CD8+ cytotoxic T lymphocyte recruitment, pro-apoptotic signaling, hyperactivity of multiple signaling pathways, and exacerbated immune inflammation in the lung, which resulted in decreased lung injury and mortality in mice with a mouse-adapted A/FM/1/47-A(H1N1) strain infection. Authors suggested that EA may have a therapeutic potential in the treatment of influenza [24].

#### 2.2.2. Anti-Inflammatory Activity of EA In Vitro and In Vivo

EA at the concentration of 100 ppm did not inhibit the activity of cyclooxygenases I and II. The authors observed that at a much lower concentration (60 ppm) the compound revealed antioxidant activity in model liposome oxidation assay, comparable to butylated hydroxyanisole and butylated hydroxytoluene, used as positive controls [25]. In another study, EA revealed anti-inflammatory activity in vitro, significantly inhibiting thrombin and neutrophil elastase activity, with IC_50_ 5 and 0.5 µM, respectively [26].

An in silico study investigated the ability of different fatty acids to bind to the PLA2 enzyme, using a molecular docking technique. Among the 46 tested fatty acids, EA revealed one of the best parameters of binding to the PLA2 protein and obtained the highest thermodynamic parameters in the isothermal titration calorimetric method. The research results indirectly explain the legitimacy of using oils rich in, e.g., EA in the treatment of rheumatoid arthritis in Ayurvedic medicine [27].

In a 60-day trial, the relationship between EA feeding and the intestinal immune function of on-growing grass carp (*Ctenopharyngodon idella*) (*n* = 24) was investigated. The different levels of EA were added to the diet: 0.00 (control), 0.29, 0.60, 0.88, 1.21, and 1.50%. The compound reduced the activities of lysozyme and acid phosphatase and the contents of complement 3 (C3), C4, and immunoglobulin M in the intestine. Additionally, EA decreased the transcript levels of liver-expressed antimicrobial peptide (LEAP)-2A, LEAP-2B, hepcidin, β-defensin-1, and mucin2. The authors also observed aggravated inflammatory response in relation to the increased transcript levels of pro-inflammatory cytokines involved in the activation of [IκB kinase β, γ (IKKβ, γ)/inhibitor of κBα (IκBα)/nuclear factor (NF)-κBp65 and c-Rel] signaling pathway and to the decreased transcript levels of anti-inflammatory cytokines involved in the suppression of [target of rapamycin (TOR)/ribosomal protein S6 kinases 1 (S6K1) and eIF4E-binding proteins (4EBP)] signaling pathway. EA did not change the transcript levels of interleukin (IL)-12p35, NF-κB p52, and IKKα in the intestine of on-growing grass carp [28].

#### 2.2.3. Cytotoxic and Anticancer Activity of EA and Its Derivatives In Vitro and In Vivo

A few studies investigated the cytotoxic potential of EA. No impact on the viability of three human breast cancer cell lines: malignant MCF-7 and MDA-MB-231 and non-malignant HBL-100 were observed at the concentration of EA up to 100 µM [29]. In other studies, EA inhibited the colony formation of human glioma C6 cells and CRL11372 osteoblasts in soft agar in vitro. For both cell lines, the concentration of 100 µM was most effective, with 53 and 25.18% of the inhibition, respectively, when compared to the control. Moreover, EA decreased the doxorubicin potential of inhibiting S-phase in glioma cells. Subsequently, the anticancer effect was also tested in vivo, by the administration of low doses (5 mg daily, *i.p*., corresponding to 250 mg/kg) of EA to mice (*n* = 16) with Ehrlich tumor extended the survival of the animals to 27 days, in comparison to 20.75 days in control, untreated group. Interestingly, higher doses of EA (20 mg, corresponding to 1 g/kg) significantly decreased the survival time to 11.7 days. The authors claimed that although the results are preliminary, the higher dose used in the pilot study may correspond to the toxic dose in humans (about 70 g) [13].

EA was also studied in terms of its combination with other drugs, to increase their cytotoxic effect. An interesting example of such use was described by Erdlenbruch et al. [30] who combined EA with compounds of the alkylphosphocholine (APC) group which show a potent anticancer activity, but also gastrointestinal toxicity at oral use as a side effect. The synthesis of erucylphosphocholine (ErPC) allowed for the intravenous administration of the drug, which resulted in a continuous increase in its concentration in serum and high accumulation in several organs, especially in brain tissue. ErPC administered in repeated doses of 10 mg/kg/day for 2 to 4 weeks did not cause serious side effects or toxicity and was well tolerated by the animals. Based on these results, it can be concluded that combining compounds from the APC group with EA may bring positive effects, including the possibility of intravenous administration of the drug and thus its use in anticancer therapy. Chrzanowska et al. [31] investigated the cytotoxic activity of ciprofloxacin and moxifloxacin conjugates with a number of fatty acids, including EA. The tests were performed on HaCaT normal cancer cells, primary and metastatic (SW480 and SW620) colon cancer cells, and PC3 metastatic prostate cancer cell line. The results showed an almost two-fold increase in the cytotoxic activity of the combination of ciprofloxacin and moxifloxacin with EA when compared to the compounds alone. However, other fatty acids used in the study were more potent [31,32]. A similar effect was observed in further studies by the same group of authors as other prostate cancers (LNCaP and DU-145) and normal (RWPE-1) cell lines. EA conjugate with ciprofloxacin showed increased, but still moderate cytotoxicity, with IC_50_ ranging from 27.6 to 73.3 µM, and 60.8 µM for cancer and normal prostate cells, respectively [32].

#### 2.2.4. Neuroprotective Activity of EA In Vivo

Two studies described the effect of EA on some neurodegenerative diseases. Kim et al. [33] evaluated the effect of EA on cognitive function or ameliorated scopolamine-induced memory impairment in normal naïve male (ICR CD-1^®^) mice (*n* = 50). EA was delivered (*p.o*.) in three doses: 1, 3, or 10 mg/kg. The medium dose (3 mg/kg) ameliorated scopolamine-induced memory impairment, as assessed via the behavioral tasks (the passive avoidance, Y-maze, and Morris water maze tasks). The administration of EA increased the phosphorylation levels of phosphatidylinositide 3-kinase (PI3K), protein kinase C zeta (PKCζ), extracellular signal-regulated kinase (ERK), cAMP response element-binding protein (CREB) and additional protein kinase B (Akt) in the hippocampus. These results suggest this effect was partially due to the activation of PI3K–PKCζ–ERK–CREB signaling as well as an increase in phosphorylated Akt in the hippocampus. Authors also indicated that EA was less effective at the high dose of 10 mg/kg, and an inverted-U-shaped dose–response curve was observed in the passive avoidance and Y-maze tasks. Results obtained suggest that high doses of EA caused the activation of muscarinic autoreceptors. It was concluded that EA may be a novel therapeutic agent for diseases associated with cognitive deficits.

As EA is peroxisome proliferator-activated receptors (PPARs)-ligand agonist, which may provide its neuroprotection, a recent study described EA effects in rotenone-induced Parkinson’s disease (PD) model in zebrafish (*n* = 60), focusing on the gut-brain axis. The animals were administered with rotenone (5 µg/L) and EA (7 µL/g) alone and in a combination of both substances. After four weeks of treatment, the locomotor activity of the fish was measured and brain and intestine tissues were further analyzed, in terms of peptides content, lipid peroxidation (LPO), nitric oxide (NO), alkaline phosphatase, superoxide dismutase, glutathione S-transferase (GST), acetylcholinesterase, and the expressions of PD-related genes. EA treatment corrected the changes in the rotenone-dysregulated expression of 196 and 243 proteins in brain and intestine samples, respectively, associated with a cytoskeletal organization, transport, and localization. Moreover, EA improved the locomotor activity of the animals and the expressions of TH, PD-related genes, and oxidant damage in the brain and intestines, which manifested as a decrease in LPO and NO and an increase in GST. Additionally, co-administration of EA and rotenone caused a significant increase in acetylcholinesterase activity in the brain and intestines, when compared to the rotenone group. Although further studies are needed in other animal PD models, the results presented seem to be in perspective [34].

#### 2.2.5. Use of EA as a Carrier for Other Drugs

Some studies described the possibility of the use of EA as a polymer delivering other drugs to improve the effectiveness of the therapy. Judy et al. [35] investigated the polymer consisting of dimeric EA and sebacic acid in a 1:1 ratio, loaded with 4-hydroperoxyclophosphamide (4HC) at the range of the concentration 0–50%, in rats (*n* = 90) implanted with 9 L gliosarcoma and F98 glioma. The minimum toxicity and the longest survival of the animals were noted for the polymer containing 20% of 4HC, while 40% of the animals survived over 80 days. Additionally, no tumor was found in the animals sacrificed at month 6 of the study. The same group of authors in their subsequent experiment assessed in vivo the efficacy and toxicity of rat F98 gliomas therapy with carboplatin, delivered by sustained-release polymers, which was to improve the poor penetration through the blood-brain barrier of the drug, and decrease its systemic toxicity. The tested FAD:SA copolymer, composed of EA dimer and sebacic acid (18:78), was loaded with increasing doses of carboplatin (0, 3, 5, 7, and 10%) and implanted into the brains of rats (*n* = 122). Next, the efficacy of the polymer containing non-toxic doses of carboplatin (1, 2 or 5%) administered intracranially was compared to the systemic administration (*i.p*.) of carboplatin (10, 30, and 50 mg/kg/week). All tested concentrations of carboplatin loaded into the polymer prolonged animal survival, but a 5% dose was the most effective. Moreover, locally administered carboplatin was more effective than its systemic administration, with reduced systemic toxicity at the same time. Thus, the use of a polymer may extend the exposure time of tumor cells to the cytotoxic drug and therefore increase the effectiveness of the therapy [36].

Golenser et al. [37] described the use of poly(FAD-SA) containing EA and sebacic acid in a 22:78 ratio as a device for slow drug release in the treatment of animal models of malaria. One of the tested drugs was desferrioxamine (DFO), a hydrophilic iron chelator that inhibits the proliferation of *Plasmodium falciparum* in vitro. The in vivo assessment was performed on mice (*n* = 30) infected with *Plasmodium vinckei petteri* by single insertion or postpatently injections of a drug-containing poly(FAD-SA) device from which the drug was released within 7 days with first-order kinetic. No side effects or toxicity were observed with drug-loaded and drug-free polymer, it was generally well-tolerated in mice. Additionally, there were no significant differences in the effect of DFO depending on the method of administration (*s.c.* or *i.p*.). The results of the above-mentioned studies show that the use of polymeric carriers composed of a combination of EA and sebacic acids, which cause a slow release of the drug, may have a positive effect on the antimalarial efficiency, especially when the effectiveness of both hydrophilic and lipophilic drugs depends on maintaining a stable blood level of the drug.

**Figure 2:**
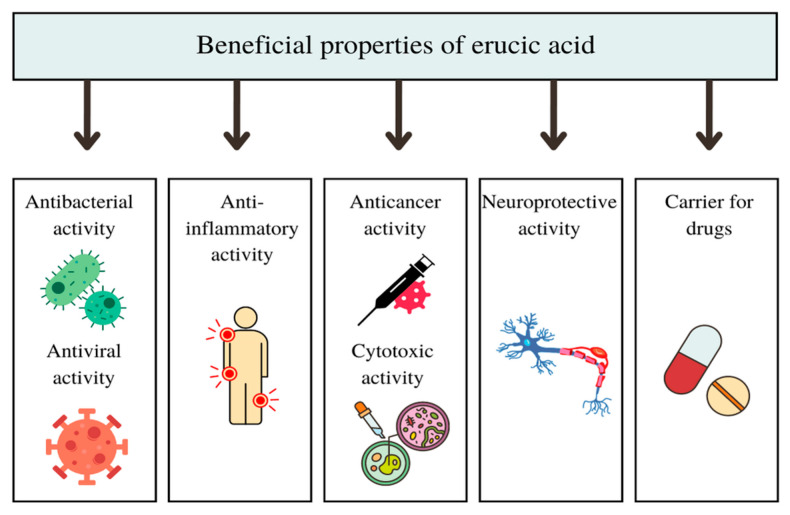
Summary of the beneficial properties of erucic acid.

A brief summary of the beneficial effects of EA is presented in Figure 2.

## 3. Materials and Methods

**Figure 3:**
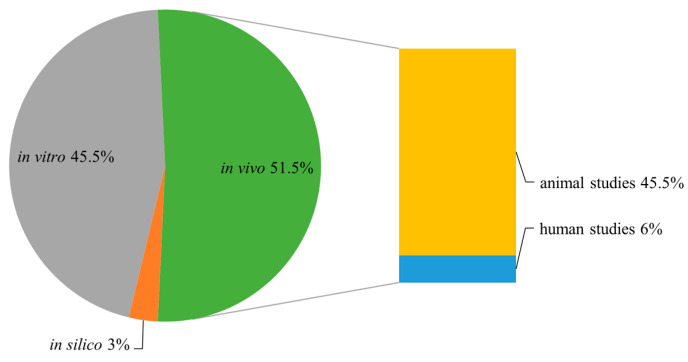
The share of different types of experiments performed with EA in the studies within this review.

The following databases were searched: Pubmed, Ovid Medline, Scopus, Web of Science, and Google Scholar, with no time limit. The publications have been selected by using the following keywords: combinations of “erucic acid”, toxic, cardiotoxic, hepatotoxic, antibacterial, antifungal, antioxidant, cytotoxic, antiviral, antiprotozoal, anti-inflammatory, antitumor, and neuroprotective. Reference lists of all articles were further checked for additional publications. The main criterion of article choice was that the experiments concerning pharmacological or biological activity were performed on erucic acid alone. The exclusion criteria were the experiments performed on (i) rapeseed oil or any other oil, with the determined amount of erucic acid; (ii) plant extracts containing erucic acid; and (iii) Lorenzo’s Oil, apart from some basic data needed as an introduction to the subject of the review. The results of the in vitro studies, in vivo experiments, and human studies were taken into consideration (Figure 3).

## 4. Conclusions and Final Remarks

Due to the unproven responsibility for the poisoning effect in TOS, and the in vivo data on inducement of cardiotoxicity, erucic acid has been for decades classified as a toxic substance, the use of which should be rather avoided. It should be strongly emphasized however, that the cardiac adverse effects have not been confirmed in humans, and the experiments in animal models, performed mostly about four decades ago, had many limitations, including a small number of animals, or extremely high doses applied during long time exposure to EA (Table 1). Moreover, this effect was reversible in most cases.

In our own opinion, the presented toxicological data clearly indicate that, when used in reasonable amounts, EA is not as toxic as it was previously believed. One of the most important issues concerns the reversibility of the potentially toxic effects of EA, after the end of the treatment. As far as the beneficial effect of EA is concerned, special attention should be paid to its role in neurodegenerative diseases, based especially on the observed ability of the compound to decrease the rate of the development of ALD. Additionally, the results of the published studies on the beneficial effects of the compound are promising enough to continue further research, despite the relatively small data presented.

According to EFSA, a tolerable daily intake (TDI) for erucic acid was established at 7 mg/kg body weight per day [2], which should be the starting point for discussing the optimal EA doses for future pharmacological experiments. Further research should be particularly directed at (i) verification of EA toxicity; (ii) cytotoxic screening of EA against a wide panel of cells of different origins; (iii) investigating the neuroprotective potential of EA in further models in vitro and in vivo; and (iv) EA use in conjugates or combined use with other drugs, as well as exploring its role as a carrier for other drugs.

## Figures and Tables

**Table 1:** Summary of the animal and human studies concerning cardiotoxicity of EA.

Treatment	Effect	Ref.
	Animal Studies	
1.4 or 2.6 g EA/100 g diet	↓ biochemical processes in heart mitochondria, reversible after the end of the treatment	[3]
5.4% EA in diet	no increase in EA level in hearts; myocardial lesions not related to EA but rather to triglyceride imbalance	[8]
5% EA in diet	impact on the metabolism of phosphatidylcholine in the heart	[10]
8.9% EA in diet	no less contractile reserve capacity effect; no epicardiac fibrotic lesions; no electrocardiographic changes	[11]
0.5 or 5% EA in diet	no changes in malondialdehyde concentration, catalase, and cytochrome c oxidase activity and isolated heart measurements; ↑ DOX toxicity in co-treatment	[12]
EA and DOX (5 mg/kg + 100 mg/kg)	↓ DOX toxicity; ↑ cardiac structure morphology	[13]
	Human Studies	
500 mL mustard oil/month	no correlation between EA intake and degenerative cardiomyopathy	[14]
prospective study	↑ level of EA in plasma correlated with ↑ risk of congestive heart failure	[15]

## Data Availability

Not applicable.
